# Interaction of Age and Mechanical Stability on Bone Defect Healing: An Early Transcriptional Analysis of Fracture Hematoma in Rat

**DOI:** 10.1371/journal.pone.0106462

**Published:** 2014-09-04

**Authors:** Andrea Ode, Georg N. Duda, Sven Geissler, Stephan Pauly, Jan-Erik Ode, Carsten Perka, Patrick Strube

**Affiliations:** 1 Julius Wolff Institute, Charité - Universitätsmedizin, Berlin, Germany; 2 Berlin-Brandenburg Center for Regenerative Therapies, Berlin, Germany; 3 Klinik für Orthopädie, Centrum für Muskuloskeletale Chirurgie, Charité - Universitätsmedizin, Berlin, Germany; National University of Ireland, Galway (NUI Galway), Ireland

## Abstract

Among other stressors, age and mechanical constraints significantly influence regeneration cascades in bone healing. Here, our aim was to identify genes and, through their functional annotation, related biological processes that are influenced by an interaction between the effects of mechanical fixation stability and age. Therefore, at day three post-osteotomy, chip-based whole-genome gene expression analyses of fracture hematoma tissue were performed for four groups of Sprague-Dawley rats with a 1.5-mm osteotomy gap in the femora with varying age (12 vs. 52 weeks - biologically challenging) and external fixator stiffness (mechanically challenging). From 31099 analysed genes, 1103 genes were differentially expressed between the six possible combinations of the four groups and from those 144 genes were identified as statistically significantly influenced by the interaction between age and fixation stability. Functional annotation of these differentially expressed genes revealed an association with *extracellular space*, *cell migration* or *vasculature development*. The chip-based whole-genome gene expression data was validated by q-RT-PCR at days three and seven post-osteotomy for MMP-9 and MMP-13, members of the mechanosensitive matrix metalloproteinase family and key players in cell migration and angiogenesis. Furthermore, we observed an interaction of age and mechanical stimuli *in vitro* on cell migration of mesenchymal stromal cells. These cells are a subpopulation of the fracture hematoma and are known to be key players in bone regeneration. In summary, these data correspond to and might explain our previously described biomechanical healing outcome after six weeks in response to fixation stiffness variation. In conclusion, our data highlight the importance of analysing the influence of risk factors of fracture healing (e.g. advanced age, suboptimal fixator stability) in combination rather than alone.

## Introduction

Due to the ageing of the population the high incidents of delayed or mal-unions after fracture trauma develops to a growing concern. The classical boundary conditions that influence healing (mechanics, surgery, accompanying traumata) are overlapped by the age-related changes in regenerative capacity.

Fracture consolidation is significantly influenced by many biological and mechanical factors. An important biological risk factor is the age of the patient. Animal experiments in rats and clinical studies in humans show a delayed course of bone healing with increasing age[1]–[3]. Possible reasons for this could be a diminished number of mesenchymal progenitor cells, their reduced migration potential and higher susceptibility towards senescence, and reduced local or systemic blood flow in older individuals [4], [5]. Inadequate fracture stability - determined by fixation stability - is the principle mechanical factor that leads to a non-union [6], [7]. An optimal mechanical stimulus enables successful fracture healing, whereas too little or too much disables it. Especially the early phase of bone healing seems to be sensitive to mechanical loading conditions [8]. In clinical cases biological and mechanical boundary conditions both jointly interact with each other and influence regeneration. The negative influence of mechanical instability on the biological factor of vascularity, endochondral ossification and maturation is an important example for this [9], [10]. Vascularity is not only disturbed by the trauma itself and/or by surgical disruption but also by (initial) instability at the fracture site[6] [11]. A failure of angiogenesis is critical, since angiogenesis is not only responsible for the oxygen supply, but also a prerequisite for the resorption of necrotic tissue and recruitment of different cell types including mesenchymal progenitor cells, which is necessary for a mechanically stable repair of the bone defect [12]. However, it remains unclear whether one stressor to regeneration - mechanical stability or age - dominates or how they interact.

In recent animal studies we provided evidence that the healing outcome of bone regeneration depends not only on mechanical stability or age alone, but more importantly, on the overlap of both stressors [1], [9]. Our data revealed a statistically significant interaction between the effects of mechanical stability and age on radiological outcome at two and six weeks and on biomechanical callus competence at six weeks post-operative. For example, in young rats, the biomechanical parameters torsional stiffness and maximum torque at failure were improved when bone defects were rigidly fixated, whereas the opposite was true for old rats [1]. However, on a more microscopically level, results were not as explicit. Micro-computed tomography could not reveal an interaction between the effects of mechanical stability and age on callus size, geometry, microstructure, and mineralization. Similar results were observed for histological analysis of vascularity and bone remodelling. Rather, we found a complex mixture of differences in the investigated parameters between the groups. For example, fixator stability influences callus size and geometry, whereas age influences callus strut thickness and perforation within these struts [9].

Gene expression in the early fracture hematoma is also known to be influenced by either age or fixation stability. For example, Meyer *et al.* found significantly lower levels of mRNA levels for Indian hedgehog and bone morphogenetic protein 2 (*BMP2*) in the fracture callus of old rats compared to young ones [13]. Comparative analysis of stabilized and non-stabilized fractures in small animals revealed differences in molecular signals controlling chondrogenesis [14]. In large animals, mRNA expression levels of members of the BMP-, tumor necrosis factor (*TNF*)- and matrix metalloproteinase (*MMP*) families as well as genes involved in bone matrix generation were lower in the critical fixation compared to the rigid fixation group at several time points [10]. However, little is known about the interaction between the effects of mechanical stability and age on gene expression. This is especially important during the early phase of bone healing, which has been shown to be mechanically sensitive and therefore crucial for the healing outcome [8].

In the present study we performed a chip-based whole-genome gene expression analysis of fracture hematoma tissue from young and old rats that underwent rigid and semi-rigid bone defect fixation. Our aim was to identify genes and, through their functional annotation, related biological processes that are influenced by an interaction between the effects of mechanical stability and age. In conclusion, we identified a number of genes and their functional annotation revealed an association with cell migration and blood vessel formation, which is so far unknown.

## Materials and Methods

### Animals and groups

All animal experiments were carried out according to the policies established by the Animal Welfare Act, the NIH Guide for Care and Use of Laboratory Animals, and the National Animal Welfare Guidelines and were approved by the local legal representative (LAGeSo Berlin, G0190/05).

Operations and postoperative care were performed according to a previously published protocol and employed a standardized biomechanically validated external fixation device [15]. Preoperatively the animal husbandry was performed in large cages (ground area 1800 cm^2^, height 19 cm, ground covered with soft-wood granule animal bedding) with a maximum of 6 animals per cage. After operation we changed to single animal husbandry in a smaller cage (ground area 810 cm^2^, height 19 cm, soft-wood granule bedding). Housing facility was specific pathogen free with a 12/12 h light/dark rhythm and a room temperature of 24°C. Animals had free access to water and food (pressed diet pellets for rodents). Preoperatively, as well as before surgical intervention for harvesting the fracture hematoma rectal temperature was measured to detect possible infections (temperature >38 °C). Postoperatively the animals were visited daily and if necessary analgesia was given. The experimental model has been previously described [1] and is briefly summarized here. For gene-chip and q-RT-PCR (day 3) analysis thirty-six and for q-RT-PCR analysis (day 7) twenty female Sprague–Dawley (Sprague-Dawley SD (Aged for old groups) Outbred rats, Harlan Laboratories, Indianapolis, USA) rats were divided into four groups with nine (day 3) respectively five (day 7) animals each group. Groups were defined by variation of fixator stabilities (rigid vs. semi-rigid) and age (12 vs. 52 weeks): young rigid (YR), young semi-rigid (YSR), old rigid (OR), and old semi-rigid (OSR). Weights of the old animals ranged at 313.4±17.4 g whereas that of the young ones was 251.9±15.3 g (*p*<0.001 in t-test). Animals were not restricted in weight bearing. In cases of adverse events (infection, major bleeding, pin loosening, implant failure or complications related to anaesthesia) the animal was sacrificed as described and a new animal was included/operated to gain the planned group sizes. Regarding this, two animals of the YSR group died for unknown reasons during primary surgery in general anaesthesia, and two aged rats (one OR, one OSR) presented with pin loosening prior to harvesting at day 7.

### Surgical procedure

**Figure 1:**
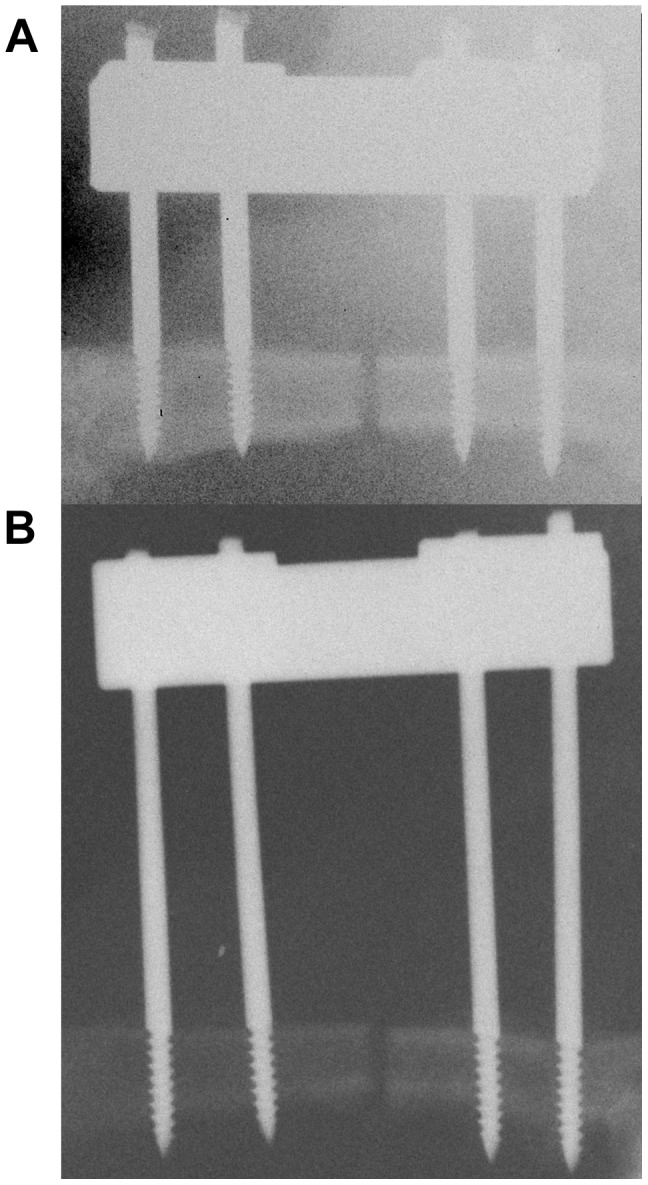
Radiographs of the two fixator configurations varying in the distance between bone and fixator crossbar (offset). (A) Rigid configuration with a 7.5 mm offset. (B) Semi-rigid configuration with a 15 mm offset. Osteotomy gap was set to 1.5 mm.

Using an anterolateral approach, the left femur was osteotomized at the midshaft, distracted to a gap of 1.5 mm and externally fixated employing a previously described fixation system [1]. The distance between fixator and bone (offset) was set to 7.5 mm in the rigid configuration (leading to a torsional 8.13 Nmm/° and axial 25.21 N/mm fixator stiffness) and 15 mm in the semi-rigid configuration (torsional 6.62 Nmm/°, axial 10.39 N/mm stiffness) (Figure 1). Before sacrifice and under general anaesthesia (see anaesthesia protocol published before [16]) the wound was reopened, inter-fragmentary fracture hematoma was harvested with a sterile forceps, directly transferred into a sterile container and frozen immediately in liquid nitrogen. For the gene-chip-analysis follow-up was three days, for q-RT-PCR analysis follow-up was three (RNA of the gene chip animals was used) and seven days. Animal sacrifice was performed in deep general anaesthesia by intracardial injection of 5 ml potassium chloride (7.45%, B.Braun Melsungen AG, Melsungen, Germany) [16].

### RNA Isolation, cDNA Synthesis, and Quantitative Reverse Transcription-Polymerase Chain Reaction

At day three and seven post-OP, total RNA was isolated from fracture hematoma by using Trizol (following the instructions of the manufacturer) starting with an initial stepwise mechanical destruction of the tissue with syringe cannulas of three different diameters until liquid was homogeneous without visible tissue particles. Next, RNA was reversely transcribed to cDNA using iScript cDNA Synthesis kit (Bio-Rad, Munich, Germany) according to the manufacturer's instructions. RNA quality was evaluated by visualizing the 18S/28S rRNA on a 1.5% agarose gel. Quantification of *MMP-2*, *MMP-9*, *MMP-13*, and *TIMP-2* were assessed by quantitative reverse transcription-polymerase chain reaction (q-RT-PCR) using the iQ SYBR Green Supermix and the iQ 5 Multicolor Realtime PCR Detection System and software (Bio-Rad, Munich, Germany) using the delta-Ct-method. The transcript expression was normalized versus the housekeeping gene β-actin (*ACTB*), elongation factor 1-alpha 1 (*EEF1A*), and glyceraldehyde-3-phosphate dehydrogenase (*GAPDH*). The primers used in the real-time PCR assay were commercially purchased (Invitrogen, Karlsruhe, Germany; Table 1). Amplification efficiency (E) was assessed to be between 1.9 and 2. At day 7, transcripts from five animals were analyzed. At day 3, analysis was performed with three pools of total RNA from three animals per pool. Each experiment was conducted in triplicates.

**Table 1:** Primer sequences.

Protein	Gene	Primer Sequence (forward/reverse)
matrix metalloproteinase 9	Mmp-9	5′ GTCTGGATAAGTTGGGGCTA 3′
		5′ GCCTTGTCTTGGTAGTGAAA 3′
matrix metalloproteinase 13	Mmp-13	5′ CAGTCTCTCTATGGTCCAGG 3′
		5′ TGGTCAAAAACAGTTCAGGC 3′
actin cytoplasmic 1 (β-actin)	Actb	5′ TGTCACCAACTGGGACGATA 3′
		5′ GGGGTGTTGAAGGTCTCAAA 3′
glyceraldehyde-3-phosphate	Gapdh	5′ ATGGGAAGCTGGTCATCAAC 3′
dehydrogenase		5′ GTGGTTCACACCCATCACAA 3′
elongation factor 1-alpha 1	Eef1a	5′ CCCTGTGGAAGTTTGAGACC 3′
		5′ CTGCCCGTTCTTGGAGATAC 3′

### Affymetrix gene chip hybridization

The amplification and labeling of the RNA samples, isolated at day three post-OP, were carried out according to the manufacturer's instructions (Affymetrix, Santa Clara, CA). Briefly, total RNA was quantified by UV-spectroscopy and its quality was checked by analysis on a LabChip (BioAnalyzer, AGILENT Technologies, Santa Clara, CA). Between one to three micrograms from each sample were synthesized into double-stranded cDNA using SuperScript transcriptase II (Life Technologies, Inc., Carlsbad, CA) and with an oligo(dT)24 primer containing a T7 RNA polymerase promoter (TIBMOL Biol, Berlin, Germany). After RNAse H – mediated (Roche, Germany) second strand cDNA synthesis, the product was purified and served as template in the subsequent in vitro transcription (IVT) reaction. Labeled complementary RNA (cRNA) was prepared from double-stranded cDNA by in vitro transcription using the GeneChip RNA transcript labeling kit (Affymetrix, Santa Clara, CA). After cleanup (Qiagen, Hilden, Germany), the biotin-labeled cRNA was fragmented by alkaline treatment [40 mmol/L Trisacetate (pH 8.2), 100 mmol//L potassium acetate, and 50 mmol//L magnesium acetate] at 94°C for 35 minutes. 15 µg of each cRNA sample was hybridized for 16 hours at 45°C to an Affymetrix Rat GeneChip Array 230 2.0. Chips were washed and stained with streptavidin-phycoerythrin using a fluidics station according to the protocols recommended by the manufacturer. Finally, probe arrays were scanned at 1.56-μm resolution using the Affymetrix GeneChip System confocal scanner 3000. Raw data were submitted to Affymetrix Expression Console software (v.1.3) to generate probe set summarization (CHP) files from feature intensity (CEL) files using PLIER algorithm. Analysis of differentially expressed genes was performed with Affymetrix Transcriptome Analysis Console (TAC) Software and PASW Statistics 18 (SPSS Inc., Chicago, USA). To conduct functional categorizing, all differentially expressed genes were submitted to the Database for Annotation, Visualization and Integrated Discovery (DAVID) V6.7 (http://david.abcc.ncifcrf.gov/) [17]. P-values were determined using EASE, followed by a Benjamini-Hochberg correction for multiple comparisons. Summary and visualization of Gene Ontology (GO) terms was performed with REVIGO (http://revigo.irb.hr/) [18]. For each condition group (YSR, YR, OSR, OR) three Affymetrix gene chip hybridizations were performed with separate pools of total RNA. Each pool comprised of total RNA from three animals. Thus, the analysis is based on total RNA from nine animals per condition group.

### MSC isolation, culture and mechanical stimulation

MSCs were isolated from bone marrow of 12 months old Sprague–Dawley rats selected by plastic adherence (Dobson et al., 1999). Dulbecco's modified Eagle's medium (DMEM) (Gibco, NY, USA) supplemented with 10% fetal calf serum (FCS) (Biochrom AG, Berlin, Germany) and 10 U/ml penicillin plus 100 µg/ml streptomycin was used as expansion medium for MSCs. Only cells from passages 2–4 were used for experiments. The bioreactor system used has been described previously [15]. Briefly, MSCs were trypsinized, and 2×106 cells in 350 µl of bioreactor medium (culture medium containing 2.4% Trasylol [Bayer, Leverkusen, Germany]) were mixed with 300 µl of fibrinogen/bioreactor medium (1∶2) mixture and 50 µl of thrombin S/bioreactor medium (1∶2) mixture (Tissucol; Baxter, Munich, Germany). This MSC/fibrinogen/thrombin mixture was placed between two spongiosa bone chips and allowed to solidify for 30 minutes at 37°C. The sandwich construct was placed into the bioreactor, and 25 ml of bioreactor medium was added. A strain of approximately 20% at a frequency of 1 Hz was applied in accordance with in vivo measurements of interfragmentary movement [19]. Mechanical loading was carried out for 72 hours. Afterwards, cells within the fibrin construct were isolated by 225 U trypsin/1 ml PBS.

### Transwell Migration Assay

Random migration (i.e. equal concentrations of bioactive molecules in both compartments) was measured by a modified Boyden chamber assay (Falk et al., 1980) using polycarbonate filters (8 µm pore size; Nunc, Wiesbaden, Germany) coated with or without Collagen I (100 µg/ml; Pure Col, Inamed Biomaterials, Fremont, U.S.), which is the most abundant extracellular protein of bones (Rossert and de Crombrugghe, 2002). MSCs (4×10∧4) were seeded onto the filters and incubated for 5 h at 37°C. Equal cell seeding was validated by an MTS test. Non-migrated cells were removed from the upper side of the filter by scraping, and remaining migrated cells were stained with 10µg/ml Hoechst-33342 (Invitrogen, Karlsruhe, Germany). The average numbers of migrated cells from five microscopic fields (1 mm×0.8 mm) per filter (0.47 cm^2^) were analysed using the NIH ImageJ software package (http://rsb.info.nih.gov/nih-image/). MSCs were isolated form three (old MSCs) and five (young MSCs) different animals followed by separate migration assays, which were performed in duplicates, i.e. two wells per group, the mean value being used for statistical analysis.

### Statistical analyses

The statistical analysis of q-RT-PCR data was performed using statistics software PASW Statistics 18 (SPSS Inc., Chicago, USA). If not stated otherwise, the influence of age and mechanical stability and their interaction on gene expression and migration were tested with a 2-tailed, 2-way Analysis of Variance (ANOVA) and posthoc Bonferroni correction. The parameters time (gene expression) and coating (migration) were set as covariates. The assumption of normality was tested using the Shapiro-Wilk normality test. For graphical presentation, results are presented in boxplots. The dark line in the middle of the boxes is the median. The box represents the interquartile range (IQR = Q3-Q1). The whiskers indicate 1.5xIQR. Outliers are circles between 1.5xIQR and 3xIQR of the quartiles. Extreme values are stars more than 3xIQR away from quartiles. For statistical analysis of chip-based whole-genome gene expression data see section 2.4 Affymetrix gene chip hybridization. The level of significance for all statistical tests was defined *p*<0.05.

## Results

The data discussed in this publication have been deposited in NCBI's Gene Expression Omnibus [20] and are accessible through GEO Series accession number GSE53256 (http://www.ncbi.nlm.nih.gov/geo/query/acc.cgi?acc=GSE53256).

**Figure 2:**
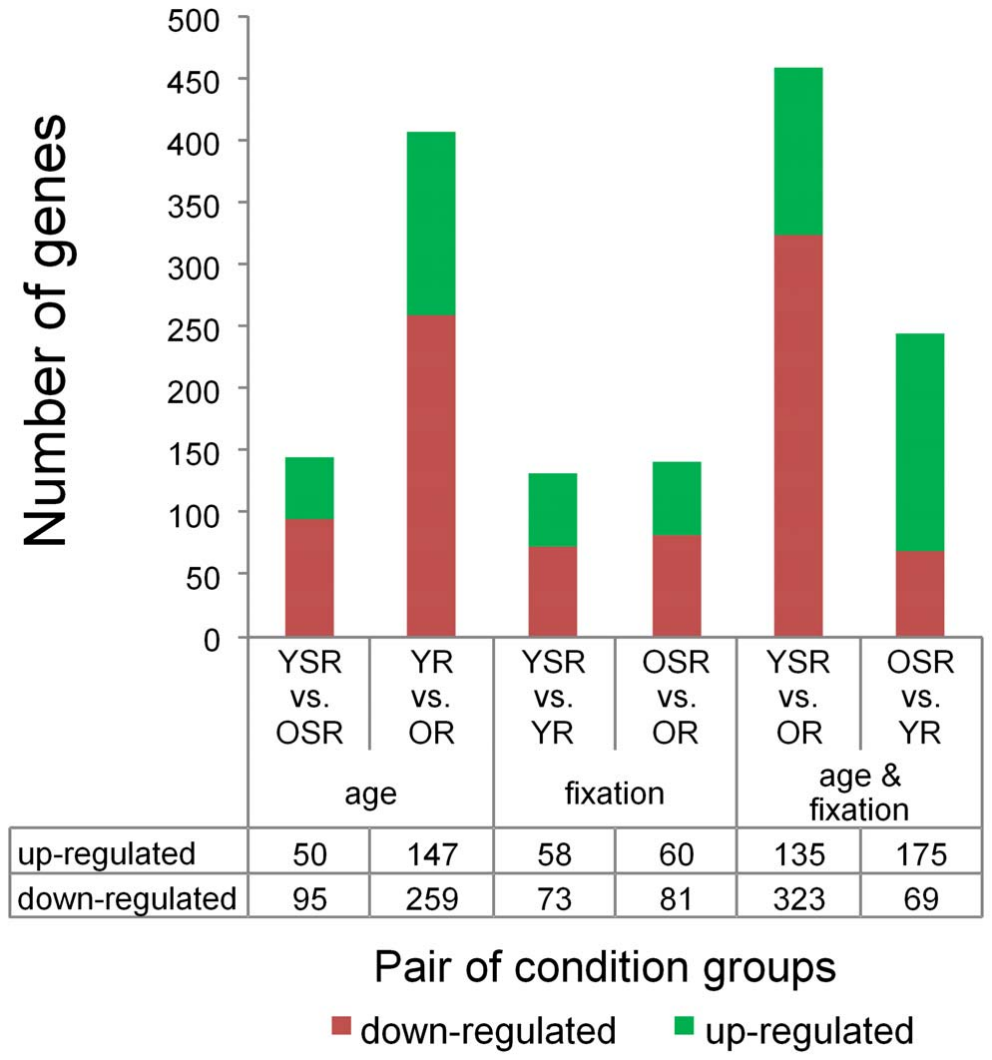
Number of differentially expressed genes for each pair of condition groups. With Affymetrix Transcriptome Analysis Console (TAC) a traditional unpaired One-Way Analysis of Variance (ANOVA) for each pair of condition groups was performed (Linear Fold Change <−2 or > 2; ANOVA p-value (condition pair) <0.05).

In total, 31099 genes were analysed with Affymetrix Transcriptome Analysis Console (TAC). TAC computes and summarizes a traditional unpaired One-Way (single factor) Analysis of Variance (ANOVA) for each pair of condition groups and for all six condition groups. In our study, 1103 genes were differentially expressed between the six possible combinations (Linear Fold Change < −2 or > 2; ANOVA p-value (condition pair) < 0.05). To illustrate the differences between the six possible combinations the numbers of up- and down-regulated genes for each pair of condition groups are displayed in Figure 2 and listed in Table S1–S6 in File S1. Since TAC cannot examine the combined effect of age and fixation stability on gene expression, a two-way ANOVA was conducted with PASW Statistics 18. However, complete analysis of 31099 genes would be computationally intensive. Therefore, 521 genes were pre-selected for analysis under the following conditions: (1) ANOVA p-value (All conditions) < 0.05 and (2) Linear Fold Change < −2 or > 2 in at least one of the six condition pairs. Statistical analysis of these 521 genes revealed a statistically significant interaction between age and fixation stability on gene expression of 144 genes (2-way ANOVA p<0.05). A complete list of these genes, the pre-selection criteria, Linear Fold Change in all six condition pairs and the results of 2-way ANOVA analysis can be found in Table S7 in File S2.

**Figure 3:**
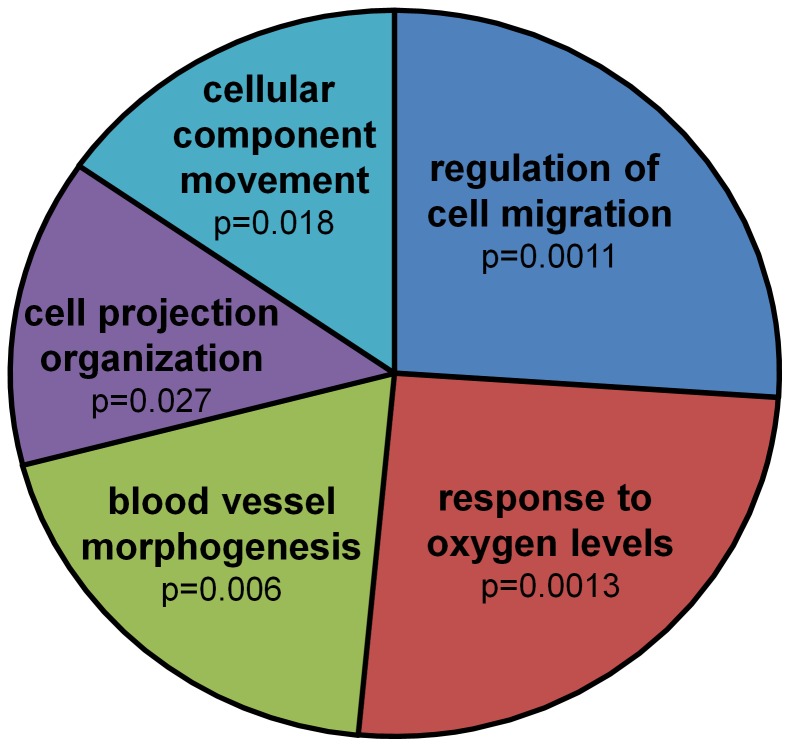
Functional annotation of 144 genes that are affected by a statistically significant interaction between age and fixation stability. Pie Chart view of REVIGO results: The single GO terms (Table S8 in File S3) are joined into clusters of related terms, visualized as sectors with different colours. Size of the sectors is adjusted (log10p-value) to reflect the p-values.

To identify biological processes related to these differently expressed genes, functional categorizing was conducted using DAVID v6.7. Functional annotation of these differentially expressed genes resulted in a list of Gene Ontology terms related to biological processes (Table S8 in File S3). Single GO terms were then joined into five clusters of related terms using REVIGO; the two dominant clusters, based on p-values, being *Regulation of cell migration* and *Response to oxygen levels* (Figure 3 and Table S9–S11 in File S4). The respective genes that were functionally categorized to these clusters by the functional annotation tool DAVID v6.7 are listed in Table 2 and 3.

**Table 2:** Gene list of cluster *Regulation of cell migration.*

Affymetrix ID	Gene Name	Linear Fold Change						p-value
		(YSR vs. YR)	(OSR vs. OR)	(YSR vs. OSR)	(YR vs. OR)	(YSR vs. OR)	(OSR vs. YR)	(2-way ANOVA)
1393403_AT	angiopoietin-like 3	−2.9	2.2	−1.5	4.1	1.4	−1.9	1.87E-02
1369814_AT	chemokine (C-C motif) ligand 20	1.2	−2.6	4.3	1.4	1.7	−3.5	2.35E-02
1370634_X_AT	chemokine (C-X-C motif) ligand 3	−3.0	−1.7	1.2	2.1	−1.4	−3.6	3.50E-02
1388459_AT	collagen, type XVIII, alpha 1	1.1	−1.8	−1.1	−2.2	−2.0	1.2	2.04E-02
1369113_AT	gremlin 1, cysteine knot superfamily, homolog (Xenopus laevis)	−1.2	−2.9	−3.2	−8.1	−9.3	2.8	4.81E-02
1369166_AT	matrix metallopeptidase 9	1.0	−5.0	−2.0	−10.4	−10.1	2.1	3.97E-03
1398275_AT	matrix metallopeptidase 9	2.0	−4.8	−1.8	−17.7	−8.7	3.7	4.67E-03
1370642_S_AT	platelet derived growth factor receptor, beta polypeptide	−1.0	−1.8	−1.1	−2.0	−2.1	1.1	2.35E-02
1393456_AT	podoplanin	−1.2	2.0	1.2	2.8	2.3	−1.4	3.90E-02
1391369_AT	serum response factor (c-fos serum response element-binding transcription factor)	−1.0	2.0	1.1	2.1	2.1	−1.1	1.22E-04
1382685_AT	slit homolog 2 (Drosophila)	1.2	−1.9	−1.1	−2.6	−2.2	1.4	4.26E-02
1392382_AT	transforming growth factor, beta 2	−1.4	1.5	−2.7	−1.2	−1.8	1.9	4.80E-02
1371240_AT	tropomyosin 1, alpha	−1.3	6.9	−1.2	7.4	5.7	−1.1	3.90E-02

Bold: Linear Fold Change <−2 or > 2.

**Table 3:** Gene list of cluster *Response to oxygen levels*.

Affymetrix ID	Gene Name	Linear Fold Change						p-value
		(YSR vs. YR)	(OSR vs. OR)	(YSR vs. OSR)	(YR vs. OR)	(YSR vs. OR)	(OSR vs. YR)	
1393902_AT	aldo-keto reductase family 1, member C1 (dihydrodiol dehydrogenase 1; 20-alpha (3-alpha)-hydroxysteroid dehydrogenase)	1.9	−3.2	1.9	−3.2	−1.7	1.0	8.82E-03
1398333_AT	endothelial PAS domain protein 1	−1.1	−3.0	−1.1	−3.0	−3.3	1.0	4.07E-02
1370604_AT	leptin receptor	1.0	−3.8	1.0	−3.8	−3.8	1.0	1.24E-02
1388204_AT	matrix metallopeptidase 13	−1.1	−4.2	−1.5	−6.0	−6.4	1.4	4.47E-02
1369166_AT	matrix metallopeptidase 9	1.0	−5.0	−2.0	−10.4	−10.1	2.1	3.97E-03
1398275_AT	matrix metallopeptidase 9	2.0	−4.8	−1.8	−17.7	−8.7	3.7	4.67E-03
1387410_AT	nuclear receptor subfamily 4, group A, member 2	1.6	−1.8	1.4	−2.1	−1.3	1.2	1.30E-03
1370642_S_AT	platelet derived growth factor receptor, beta polypeptide	−1.0	−1.8	−1.1	−2.0	−2.1	1.1	2.35E-02
1393456_AT	podoplanin	−1.2	2.0	1.2	2.8	2.3	−1.4	3.90E-02
1368170_AT	solute carrier family 6 (neurotransmitter transporter, GABA), member 1	1.0	2.8	−2.8	1.0	1.0	2.8	3.70E-05
1392382_AT	transforming growth factor, beta 2	−1.4	1.5	−2.7	−1.2	−1.8	1.9	4.80E-02

Bold: Linear Fold Change < −2 or > 2.

**Figure 4:**
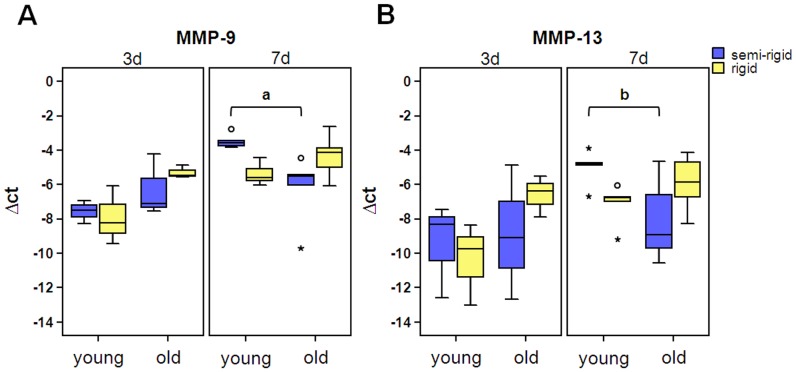
Gene expression of MMP-9 and -13 were significantly affected by the interaction of fixation and age. The expression of mRNA of YR, YSR, OR and OSR was evaluated by quantitative qRT-PCR, normalized for the housekeeping genes (HKG) *Actb*, *Gapdh*, and *Eef1a* and quantified using the delta-Ct-method: Δct  =  ct (geo mean HKG) - ct (gene of interest). (day 3, n = 3 RNA pools á 3 animals; day 7, n = 5; a, *p* = 0.019; b, *p* = 0.057; °, outlier; *, extreme value)

Interestingly, among the genes listed in Table 2 and 3 with a noticeable Linear Fold Change in gene expression are members of the family of matrix metalloproteinases (MMPs): MMP-9 and MMP-13. To validate the results of Affymetrix chip-based whole-genome gene expression analyses, the expression level of MMP-9 and -13 were analysed via qRT-PCR at day 3 and also day 7. There was also a statistically significant interaction between the effects of fixation and age on gene expression of MMP-9 (p = 0.009) and MMP-13 (p = 0.016). MMP-9 and -13 expression is also influenced by time (MMP-9, p = 0.001; MMP-13, p = 0.007). Thus results are presented separately for day 3 and day 7 (Figure 4). Post-hoc inter-group comparison revealed that MMP-9 expression is significantly higher in YSR than in OSR (p = 0.019) at day 7; for MMP-13 a trend was observed between YSR and OSR (p = 0.057).

**Figure 5:**
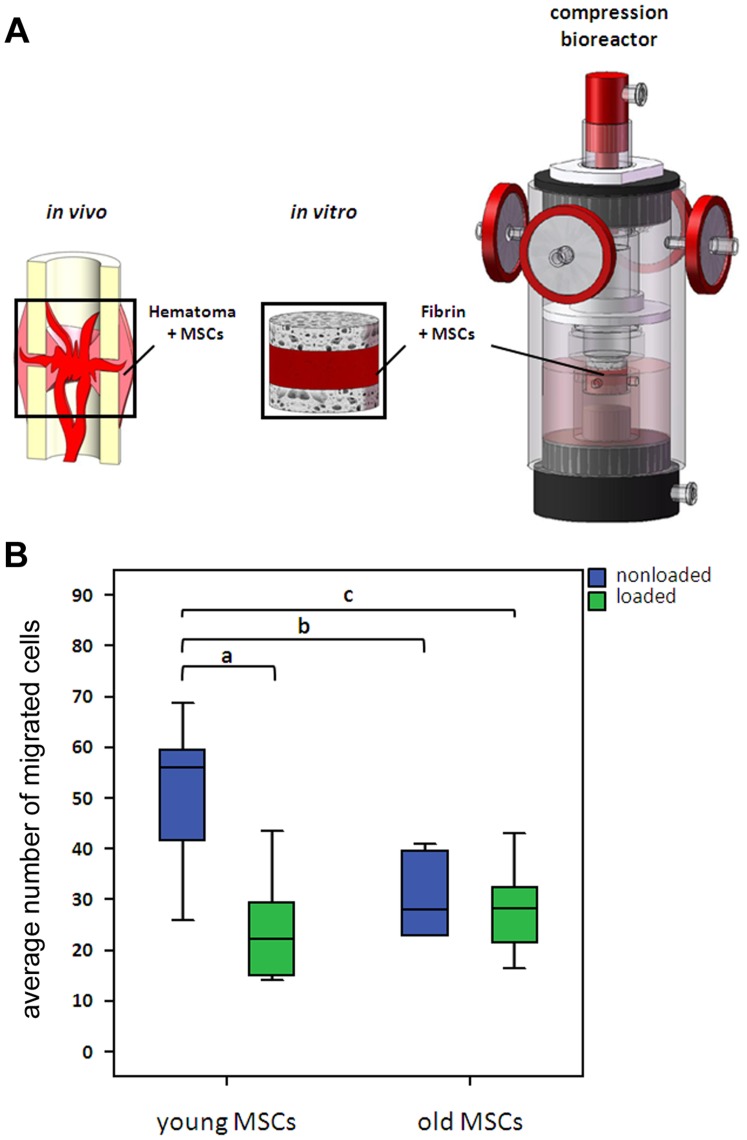
Migration of mechanically stimulated young and old MSCs. (A) Experimental set-up to investigate the effect of mechanical loading of MSC/fibrin constructs. MSCs were embedded in fibrin, placed between two cancellous bone chips and mechanically stimulated. (B) MSC migration was investigated in a modified Boyden-Chamber assay. The average number of migrated cells from five microscopic fields per filter was analysed using NIH ImageJ software. (young MSCs, n = 10; old MSCs, n = 6, a, p<0.001; b, p = 0.005; c, p = 0.003).

**Figure 6:**
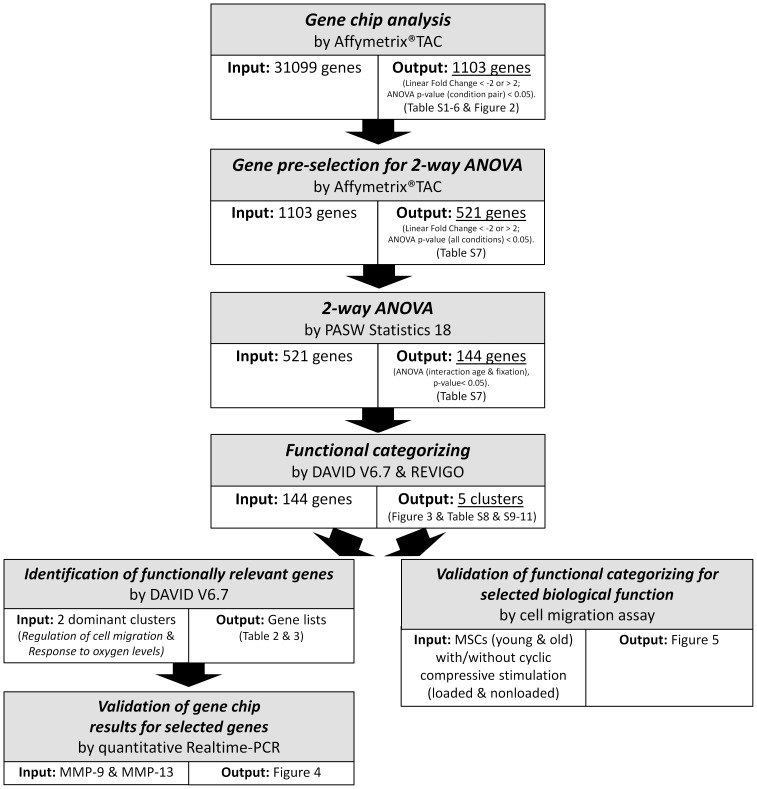
Summary and overview of the work flow in this study.

Functional annotation clustering of the 144 genes that were influenced by a significant interaction between age and fixation stability revealed the cluster *Regulation of cell migration* having the most significant p-value. To validate whether this biological process is influenced by a significant interaction between age and mechanical stability, we investigated this parameter in a simplified *in vitro* model. In order to do so, we mimicked the early phase fracture gap conditions by embedding mesenchymal stromal cells (MSCs) in fibrin, the major extracellular matrix of the hematoma, and by stimulating these cells with cyclic-compressive loading. MSCs were chosen as cell source, because they are known to be mechanosensitive and key players in bone regeneration, and they are present in the fracture hematoma by day three post-fracture [21]–[23], the same day we harvested the hematoma tissue for gene expression analyses. The MSCs were isolated from young (10–12 weeks; data published in [24]) and old (12 months) rats (yMSCs and oMSCs, respectively) in accordance with the *in vivo* experiments. To apply two different mechanical loading regimes to MSCs, similar to the ones *in vivo* (semi-rigid and rigid fixation allowing more or less interfragmentary movement, respectively), the cells underwent cyclic compression in a bioreactor (loaded) and were compared to nonloaded controls. Although the loading regimes *in vivo* (more or less movement) and *in vitro* (cyclic or no compression) did not perfectly match, the migratory behaviour of MSCs was found to be influenced by the interaction between age and mechanical stimulation (p = 0.007). Coating of the migration filter with collagen I had no influence on the results compared to no coating (p = 0.942). Thus results are displayed together (Figure 5). Inter-group comparison revealed that migration of nonloaded yMSCs is significantly higher compared to loaded yMSCs (p<0.001), nonloaded oMSCs (p = 0.005) and loaded oMSCs (p = 0.003). No statistical significance was observed between the latter three groups. A summary of the work flow in this study is given in Figure 6.

## Discussion

This study is a follow-up of two previous ones [1], [9]. Our aim in this study was to compare gene expression in fracture hematoma tissue of young and old rats that underwent either rigid or semi-rigid fixation. By using Affymetrix chip-based whole-genome gene expression analyses our aim was to identify genes and related biological processes that are influenced by an interaction between the effects of mechanical stability and age. By this we wanted to gain insights into the early hematoma's biological processes that led to the previously reported biomechanical long-term outcomes in bone defect healing, which were influenced by age and varying fixator configurations, i.e. fixation stiffness. In total, we had four experimental groups: young semi-rigid (YSR), young rigid (YR), old semi-rigid (OSR) and old rigid (OR).

The majority of fractures heal by secondary, or indirect, fracture healing involving callus formation [22]. This process is both spatially and temporally regulated [25]. By using a model of experimental fracture healing in the rat the healing cascade has been elucidated [21]. Compared to human fractures, the rat fracture healing cascade proceeds at about twice the speed [23]: Initially, bone fracture is accompanied by disruption of bone marrow, bone matrix, blood vessels, and surrounding soft tissue. Within the first 24 hours, bleeding of these tissues and releasing of bone marrow into the fracture gap give rise to the initial hematoma [26]. Degranulating platelets and inflammatory cells release cytokines and growth factors that induce migration of MSCs from bone marrow and acute inflammatory cells and further aggregation of platelets. From day two to six, in the area between the cortices, soft callus begins to form via endochondral ossification, where MSCs begin to proliferate by day three [21]–[23].

In our study fracture hematoma tissue was harvested at three days post-osteotomy. From 31099 analysed genes, 1103 genes were differentially expressed between the six possible combinations and from those 1103 genes 521 genes were selected to be analysed with a two-way ANOVA. In total, 144 genes were identified as statistically significantly influenced by the interaction between age and fixation stability. Functional annotation of these genes revealed an involvement in cell migration. Thus far, there is no data published that report on the interaction of age and mechanical stimulation on cell migration *in vivo*.

We have two possible explanations for the observations in our study: either (1) the cell type composition in the fracture hematoma of young and old animals varies so that different cells respond differently to mechanical stimulation or (2) the cell type composition is similar, but the ability of these cells to sense and adapt to mechanical stimulation has changed during aging. We recently reported that migration of young MSCs, key players in bone regeneration and present in the fracture hematoma, is reduced *in vitro* if the cells underwent mechanical stimulation compared to non-stimulated controls [24]. Therefore, we now compared migration of young and old MSCs in response to cyclic-compressive loading. Interestingly, a statistically significant interaction between the effects of mechanical stimulation and age on MSC migration could also be observed in this setting. These results point towards a reduced ability of MSCs to sense and/or adapt to mechanical stimulus with advanced age. Based on several studies, it was proposed that fracture hematoma and bone tissue of old individuals is less responsive to mechanical stimulation than that of young ones. For example, an obvious growth of the loaded tibia was reported in young but not in old animals [27]. And a higher mechanical loading threshold was needed for initiation of bone growth during remodelling in old compared to young rats [28]. We recently reported that mechanical loading *in vitro* stimulates the paracrine pro-angiogenic capacity of MSCs and human fracture hematoma [29], [30]. Interestingly, in the latter study, the angiogenic regulator *vascular endothelial growth factor* (VEGF) was up-regulated in hematoma of young but not old patients in response to mechanical loading.

The chip-based whole-genome gene expression analysis revealed that members of the family of matrix metalloproteinases (MMPs), MMP-9 and -13, were strongly influenced by an interaction of the effects of age and mechanical stability. Therefore, these results were further validated by q-RT-PCR with similar outcome. MMPs degrade most components of the extracellular matrix (ECM), such as aggrecan, collagens, elastin, or vitronectin, as well as many non-ECM molecules. Thereby, MMPs allow cell migration, participate in cleavage or release of biologically active molecules and regulate cellular behaviour such as cell attachment, growth, differentiation, and apoptosis [31], [32]. During successful enchondral ossification, MMP-9 and -13 play an important role in the fracture callus [33]. They coordinate not only cartilage matrix degradation, but also the recruitment and differentiation of endothelial cells, osteoclasts, chondroclasts and osteoprogenitors. Lack of MMP-9 in mice results in non-unions and delayed unions of their fractures caused by persistent cartilage at the injury site [34]. MMP-13-null mice showed profound defects in growth plate cartilage [35]. Several studies provided evidence for the regulation of MMP mRNA by mechanical loading *in vitro*. For example, mRNA level of MMP-9 increased as early as 3-6 h after the application of cyclic tensile load on cultured chondrocytes isolated from young rabbits [36]. MMP-13 mRNA up-regulation has been described after stretching of murine osteoblasts [37]. This is the first study that describes the simultaneous influence of age and mechanical stimuli on their expression. However, it is known that MMP function is spatially and temporally regulated at transcriptional, post-transcriptional, and post-translational levels via MMP controlled activation, inhibition and cell surface localization. Therefore, it is now crucial to validate these results also on protein level.

In summary, our results indicate that cellular migration is differently affected by fixator stability in young and old rats three days post-osteotomy possibly leading to the previously reported biomechanical long-term outcomes in bone defect healing. In conclusion, our data highlight the importance of analysing the influence of risk factors of fracture healing (e.g. advanced age, suboptimal fixator stability) in combination rather than alone.

## Supporting Information

File S1 **Table S1–S6.** Up- and down-regulated genes for each pair of condition groups identified by Affymetrix Transcriptome Analysis Console (TAC)
(XLSX)

File S2 **Table S7.** Genes that are influenced by the interaction between age and fixation stability identified by 2-way ANOVA.
(XLSX)

File S3 **Table S8.** Functional annotation of differentially expressed genes using DAVID v6.7.
(XLSX)

File S4 **Table S9–S11.** Single GO terms were joined into five clusters of related terms using REVIGO.
(XLSX)
